# Umbilical Vein Blood Flow in Uncomplicated Pregnancies: Systematic Review of Available Reference Charts and Comparison with a New Cohort

**DOI:** 10.3390/jcm12093132

**Published:** 2023-04-26

**Authors:** Moira Barbieri, Giulia Zamagni, Ilaria Fantasia, Lorenzo Monasta, Leila Lo Bello, Mariachiara Quadrifoglio, Giuseppe Ricci, Gianpaolo Maso, Monica Piccoli, Daniela Denis Di Martino, Enrico Mario Ferrazzi, Tamara Stampalija

**Affiliations:** 1 Department of Mother and Neonate, Institute for Maternal and Child Health IRCCS “Burlo Garofolo”, 34100 Trieste, Italy; moira.barbieri@unimi.it (M.B.);; 2 Clinical Epidemiology and Public Health Research Unit, Institute for Maternal and Child Health IRCCS “Burlo Garofolo”, 34100 Trieste, Italy; 3 Department of Medicine, Surgery and Health Sciences, University of Trieste, 34100 Trieste, Italy; 4 Department of Mother, Child and Neonate, Fondazione IRCCS Ca’ Granda Ospedale Policlinico di Milano, 20100 Milan, Italy; 5 Department of Clinical and Community Sciences, University of Milan, 20100 Milan, Italy

**Keywords:** umbilical vein blood flow volume, reproducibility, Doppler ultrasound, reference ranges, umbilical cord, fetus, nutrition, oxygenation

## Abstract

The objectives of the study were (1) to perform a systematic review of the available umbilical vein blood flow volume (UV-Q) reference ranges in uncomplicated pregnancies; and (2) to compare the findings of the systematic review with UV-Q values obtained from a local cohort. Available literature in the English language on this topic was identified following the PRISMA guidelines. Selected original articles were further grouped based on the UV sampling sites and the formulae used to compute UV-Q. The 50th percentiles, the means, or the best-fitting curves were derived from the formulae or the reported tables presented by authors. A prospective observational study of uncomplicated singleton pregnancies from 20^+0^ to 40^+6^ weeks of gestation was conducted to compare UV-Q with the results of this systematic review. Fifteen sets of data (fourteen sets belonging to manuscripts identified by the research strategy and one obtained from our cohort) were compared. Overall, there was a substantial heterogeneity among the reported UV-Q central values, although when using the same sampling methodology and formulae, the values overlap. Our data suggest that when adhering to the same methodology, the UV-Q assessment is accurate and reproducible, thus encouraging further investigation on the possible clinical applications of this measurement in clinical practice.

## 1. Introduction

The umbilical vein blood flow volume (UV-Q) reflects the amount of metabolites and oxygen delivered to the fetus [1]. An adequate UV-Q is essential to guarantee fetal needs for oxidative metabolism and growth [2]. The UV-Q increases progressively and exponentially throughout pregnancy, from 63 mL/min at 20 weeks to 373 mL/min at 38 weeks [3]. However, UV-Q normalized for estimated fetal weight (UV-Q/EFW) shows a progressive reduction in relation to the increasing fetal mass [4]. This suggests a progressive mismatch between fetal demands and placental availability, suggesting a possible role for UV-Q in clinical settings [5]. In fact, studies have shown a reduced UV-Q and UV-Q/EFW in fetal growth restriction (FGR) [6,7]. Lower UV-Q values have also been found in normally grown fetuses that experienced intrapartum distress [8,9], suggesting a potential role for UV-Q as an admission test [10]. The first reports regarding UV-Q measurement in human fetuses go back to the early 1980s [11,12]. Despite a great interest over the past four decades in the possibility of assessing the blood flow delivery to the fetus, this biophysical assessment has not gained ground, and it is still used only in research settings [13,14]. One of the main reasons is related to the questions raised regarding the accuracy, reproducibility, and technical aspects of the UV-Q measurement [15,16]. Over time, doubts about quantitative inaccuracies have been challenged [17,18], thanks to the improvement of ultrasound machines and the introduction of high-resolution ultrasound probes. Although UV-Q calculation has shown moderate to good intra- and inter-observer reproducibility [19], arguments against the accuracy and reproducibility of UV-Q measurement are still limiting its possible clinical use. On this ground, we performed a systematic review of the available reference ranges of UV-Q in the human fetus. We also prospectively recruited a cohort of uncomplicated singleton pregnancies between 20^+0^ and 40^+6^ weeks of gestation and performed UV-Q measurements with the aim to compare the obtained values with the results of this systematic review.

## 2. Materials and Methods

### 2.1. Systematic Review of the Available UV-Q Reference Ranges

A comprehensive systematic review was performed to identify studies that evaluated UV-Q in low-risk pregnancies. The study was registered with the International Prospective Register of Systematic Reviews database (PROSPERO registration number: CRD42021276868) [20]. The Preferred Reporting Items for Systematic Reviews and Meta-analysis (PRISMA) guidelines [21] were followed in the review report.

#### 2.1.1. Study Identification and Selection

A systematic literature search in the English language was conducted from inception until December 2021 in PubMed (Medline) and Scopus. The search strategy consisted of relevant Medical Subject Headings (MeSH) terms and keywords, including “umbilical vein blood flow”/“umbilical venous blood flow” and “volume”. Inclusion criteria were studies focusing on UV-Q values in singleton pregnancies without congenital abnormalities that were conducted in hospital settings. Study protocols, case reports, animal experimental studies, in vitro studies, review articles, editorials, letters to the editor, and conference proceedings/posters that did not appear as full-text papers were excluded. We aimed to identify studies that reported the algorithm for the calculation of the central values for UV-Q and/or UV-Q/EFW. Methods to plot the 50th percentile or the best-fitting curves were derived from the formulae presented in the manuscripts or from the reported tables of percentiles, when available. An absence of a central value for each week of gestation disqualified a study from further assessment. The following data were extracted: authors, year of publication, study type, number of participants, gestational age, type of population, sampling site of the UV, equation, and percentiles and mean values of UV-Q and UV-Q/EFW. Relevant articles were searched manually to identify manuscripts not obtained from the research strategy. The assessment of study eligibility, methodological quality, and data extraction of the included studies were completed by two independent investigators. Data from each eligible study were extracted without modification of the original information onto custom-made data collection forms. Disagreements were resolved by consensus with a third reviewer. 

A distinction between uncomplicated pregnancies and unselected or mixed high- and low-risk populations was performed. To improve the synthesis and understanding of the different issues on this topic, selected original articles were further grouped based on:

- The sampling site: (a) studies investigating UV-Q on the intra-abdominal (IA) portion of the UV; and (b) studies investigating UV-Q at the free-floating (FF) portion of the UV;
- The formula used to compute the UV-Q.

#### 2.1.2. Quality Assessment

The quality assessment of each included study was performed using the Quality Assessment of Diagnostic Accuracy Studies (QUADAS-2) criteria [22] in four domains related to the risk of bias: patient selection; index test; reference standard; and flow and timing. Each domain was categorized as “low risk”, “high risk”, or “some concerns” of bias if the data regarding the domain were “reported and adequate”, “reported but inadequate”, or “not reported”, respectively. The first three domains were assessed in respect to applicability. The overall judgement was then established based on the rating of individual domains. The robvis tool web app [23] was then used to visualize the risk-of-bias after applying the separate quality criteria.

### 2.2. Prospective Cohort Study on UV-Q, UV-Q/AC, and UV-Q/EFW

A prospective cross-sectional monocentric observational study of singleton low-risk uncomplicated pregnancies from 20^+0^ to 40^+6^ weeks of gestation was conducted to obtain reference ranges for UV-Q, UV-Q/AC, and UV-Q/EFW. The study protocol was approved by the local Ethics Committee (CEUR-2019-EM-225). Criteria for inclusion were first-trimester dating based on crown-rump length measurement, low-risk singleton uncomplicated pregnancy, and compliance with the study protocol. Exclusion criteria were twin pregnancies, premature rupture of membranes, signs of pathological obstetric condition, pregnancies complicated by the fetal structure, chromosomal abnormalities, or intrauterine infections. Eligible women were consecutively allocated to an additional ultrasound examination > 20 weeks of gestation for UV-Q, UV-Q/AC, and UV-Q/EFW measurements, together with fetal biometry and Doppler velocimetry. UV-Q, UV-Q/EFW, and UV-Q/AC were calculated as already reported [24]. We planned to recruit at least 20 women for each gestational age group. Each woman was considered once and allocated to a biweekly gestational age group (20–21; 22–23; etc.). Fetal biometry and Doppler velocimetry were performed following the International Society of Ultrasound in Obstetrics and Gynecology guidelines [25,26]. The EFW was calculated by using the Hadlock formula [27]. UV-Q, UV-Q/AC, and UV-Q/EFW were calculated, blinded to the physician, and plotted and compared to the UV-Q and UV-Q/EFW values from other manuscripts considered eligible for this systematic review.

#### Statistical Analysis

For each variable of interest and for each gestational week, data points > Q3 + 3 × IQR (Interquartile Range) were identified as outliers and removed from the analysis. Centile curves were constructed using Generalized Additive Models for Location, Scale and Shape (GAMLSS) with the Box–Cox power exponential distribution (BCPE) or the Box–Cox Cole and Green distribution (BCCG) specified for the considered variables. Cubic or penalized splines with different degrees of freedom were used to model the scale and shape parameters. Different models were estimated for each variable using a combination of distributions and splines and the best model was selected, i.e., the model with the lowest value of the Akaike’s Information Criterion (AIC). UV-Q was modelled using penalized splines with 1 d.f. for σ, 1 d.f. for τ, and 2 d.f. for η. For UV-Q/EFW, cubic splines were used to model the scale and shape parameters, with 2 d.f. for σ, 1 d.f. for τ, and 2 d.f. for η. Cubic splines were also used in the model for UV-Q/AC, with 1 d.f. for σ, 1 d.f. for τ, and 1 d.f. for η. The distances between the estimated central curves were calculated in terms of z-scores, as proposed by DeVore et al. [28], where:

$$z=\frac{value\;from\;published\;study-predicted\;value\;from\;current\;study}{predicted\;SD\;from\;current\;study}$$

Z-score values between −1 and 1 were considered not significantly different [29]. The statistical analyses were conducted using the software R Core Team (2020) [30].

A calculator that allows UV-Q and UV-Q/EFW computation as well as the respective z-score and percentile for a specific gestational week age, by using our data as the reference point, is provided at the following webpage https://giuliazamagni.shinyapps.io/UV_Calculator/ (accessed on 7 August 2022).

## 3. Results

### 3.1. Systematic Review of the Available UV-Q Reference Ranges

**Figure 1:**
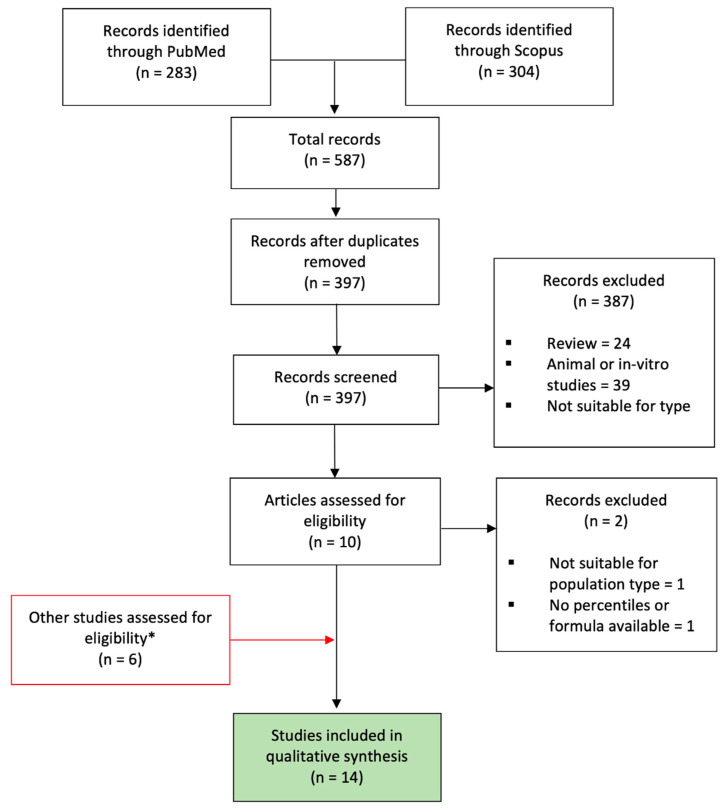
PRISMA flow diagram of the study selection. * Not identified through Pubmed or Scopus search strategy but cited in other articles similar to this systematic review or found by evaluating the bibliography of studies obtained from the research strategy. In red, the articles not found through the search strategy; in green, the articles selected for this systematic review.

The research identified 587 publications (Table S1). After the removal of duplicates, a total of 397 studies were obtained. Figure 1 shows the PRISMA flow diagram of the study selection.

Reviews, animal studies, and in vitro studies were excluded, as well as all articles that were not suitable for study type (i.e., conference proceedings, book chapters) or not suitable for the topic (not related to the research). This left 10 articles that were assessed to be eligible. A full-text review of the 10 articles excluded two additional manuscripts that were considered not suitable for the population type (Widnes et al. [31] investigated gestational age-specific serial changes in UV-Q, establishing sex-specific reference ranges) or because percentiles and/or equations were not provided by the authors (Lees et al. [32]). The evaluation of the bibliographies of the included studies further added six articles that were considered suitable for evaluation. Thus, fourteen manuscripts were included in the systematic review: eight articles [18,33,34,35,36,37,38,39] found with the research strategy and six articles [3,28,40,41,42,43] found by evaluating the bibliographies of studies obtained from the research strategy.

#### 3.1.1. Risk of Bias within Studies According to QUADAS Criteria

Figure S1 shows the assessment of the included studies by QUADAS-2 criteria. In the “patient selection” domain, two studies [37,38] were classified as having a high risk of bias because fetuses with growth impairments or women with high-risk pregnancies were also included. The remaining 12 studies were considered to have a low risk of bias. In the “index domain”, five studies [34,39,41,42,43] were classified as having ‘some concerns’ because it was not explicit if any action had been taken to test the inter- and intra-observer variability. The remaining nine studies were considered as low-risk. In the “reference range” domain, one study [3] was classified as having a high risk of bias because the exponential curve formula of the UV-Q reported by the authors did not correspond to the reported values. The remaining 13 studies were considered as low-risk. In the “flow and timing” domain, one study [36] was classified as having a high risk of bias because of its small sample size (32 low-risk pregnant women). The remaining 13 studies were considered as having a low risk of bias.

#### 3.1.2. Description of the Included Studies

The main characteristics and results of the 14 studies are summarized in Table 1. One study [38] recruited women retrospectively, while all others were prospective. Eleven studies [3,18,34,36,37,38,39,40,41,42,43] published reference algorithms for the central values of UV-Q. For the remaining three articles [28,33,35], UV-Q values were derived from the percentile data. Six studies [3,18,28,33,36,37] investigated UV-Q at the FF portion of the umbilical vein, six studies [34,35,38,40,41,43] at the IA portion, and two studies [39,42] both at the FF and IA portions of the UV. For one study [3], the formula for the UV-Q exponential curve reported by authors did not correspond to the exponential values. For this reason, we decided to exclude the study from the comparison. On the contrary, the UV-Q/EFW values were considered plausible and therefore were included in the analysis. UV-Q and UV-Q/EFW central values were not reported homogeneously. In three studies [34,36,38], the central values of UV-Q and UV-Q/EFW of the entire observational period were reported. In six studies [28,39,40,41,42,43], the central UV-Q and UV-Q/EFW values were not reported, while Sutton et al. [18] reported only UV-Q/EFW values. The remaining studies [3,33,35,37] provided UV-Q and UV-Q/EFW values, considering the gestational period at the enrolment and at the end of observation separately. Gestational time intervals and the mean values are reported in Table 1.

### 3.2. Prospective Cohort Study on UV-Q

We recruited 277 women, and of those, 12 were excluded due to an onset of pregnancy complications. This left a total of 255 low-risk women from 20^+0^ to 40^+6^ gestational weeks for UV-Q, UV-Q/EFW, and UV-Q/AC calculation. Demographic, obstetric, and neonatal characteristics of the cohort are shown in Table 2.

Based on this cohort, we established the 5th, 10th, 50th, 90th, and 95th percentiles for UV-Q, UV-Q/EFW, and UV-Q/AC for each gestational week, both combined (Table 3) and sex-specific (Table S2).

**Figure 2:**
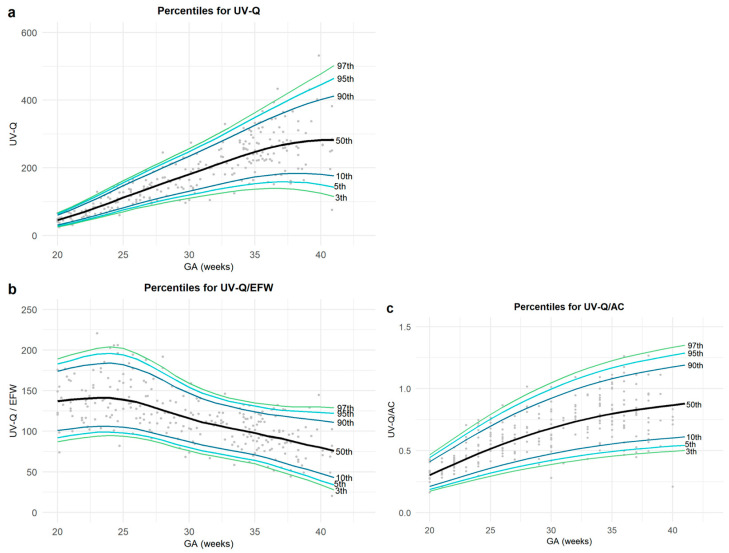
The figure represents the percentiles for (**a**) umbilical vein blood flow volume (UV-Q); (**b**) UV-Q normalized for estimated fetal weight (UV-Q/EFW); and (**c**) UV-Q normalized for abdominal circumference (UV-Q/AC).

Overall, there was an increase in UV-Q and UV-Q/AC, while UV-Q/EFW showed a decreasing trend (Figure 2).

#### Comparison between Reference Range Values for UV-Q and UV-Q/EFW

**Figure 3:**
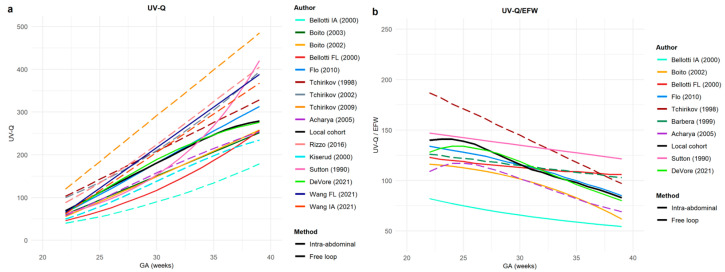
The figure represents (**a**) absolute and (**b**) normalized-for-estimated-fetal-weight (EFW) umbilical vein blood flow central values (UV-Q) from studies included in systematic review and our set of data. Straight lines (−): studies investigating UV-Q at the intra-abdominal portion of the umbilical cord; dashed lines (- -): studies investigating UV-Q at the free-floating portion of the umbilical cord. Different colors represent the first author’s name and the year of publication [18,28,33,34,35,36,37,38,39,40,41,42,43].

A comparison was performed for the 14 manuscripts included in the systematic review (Table 1) and the 15th set of data represented by our local cohort. Because the examined gestational age interval was heterogeneous among the studies (Table 1), we decided to consider a gestational age interval common to all studies (i.e., from 22^+0^ to 39^+0^ gestational weeks). Since neither crude data nor confidence intervals were available to perform a statistical comparison, all estimated curves were superimposed on a single plot in order to detect graphical differences in relation to the gestational age (Figure 3).

The comparison among UV-Q and UV-Q/EFW central curves, including low-risk and unselected populations both on IA and FF, is represented in Figures S2 and S3, while each author’s UV-Q and UV-Q/EFW central values are reported in relation to the gestational age in Tables S3 and S4, respectively.

**Figure 4:**
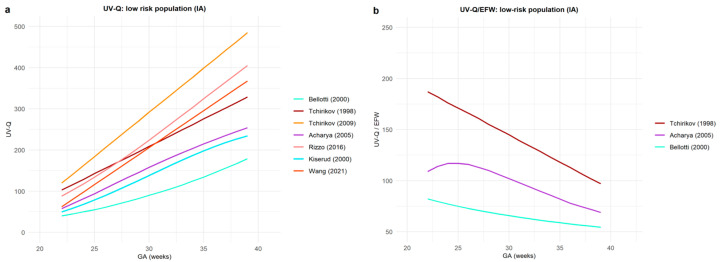
This figure represents umbilical vein blood flow volume (UV-Q) central values in low-risk populations in studies that investigated UV-Q in the intra-abdominal (IA) portion of the umbilical vein: (**a**) UV-Q absolute value; and (**b**) normalized for estimated fetal weight (UV-Q/EFW). Different colors represent the first author’s name and the year of publication [34,35,39,40,41,42,43].

**Figure 5:**
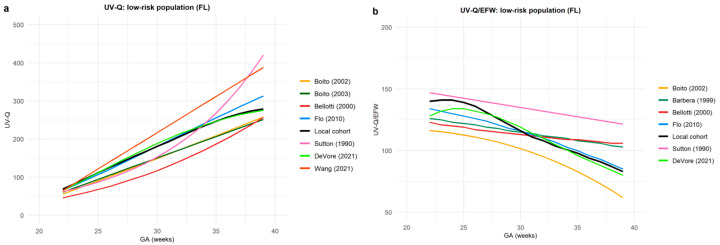
This figure represents umbilical vein blood flow volume (UV-Q) central values in low-risk populations in studies that investigated UV-Q on the free-floating (FF) portion of the umbilical cord: (**a**) UV-Q absolute value; and (**b**) normalized for estimated fetal weight (UV-Q/EFW). Different colors represent the first author’s name and the year of publication [18,28,33,36,37,39,42].

Figure 4 and Figure 5 represent UV-Q and UV-Q/EFW central values only in low-risk populations according to the UV sample site.

**Figure 6:**
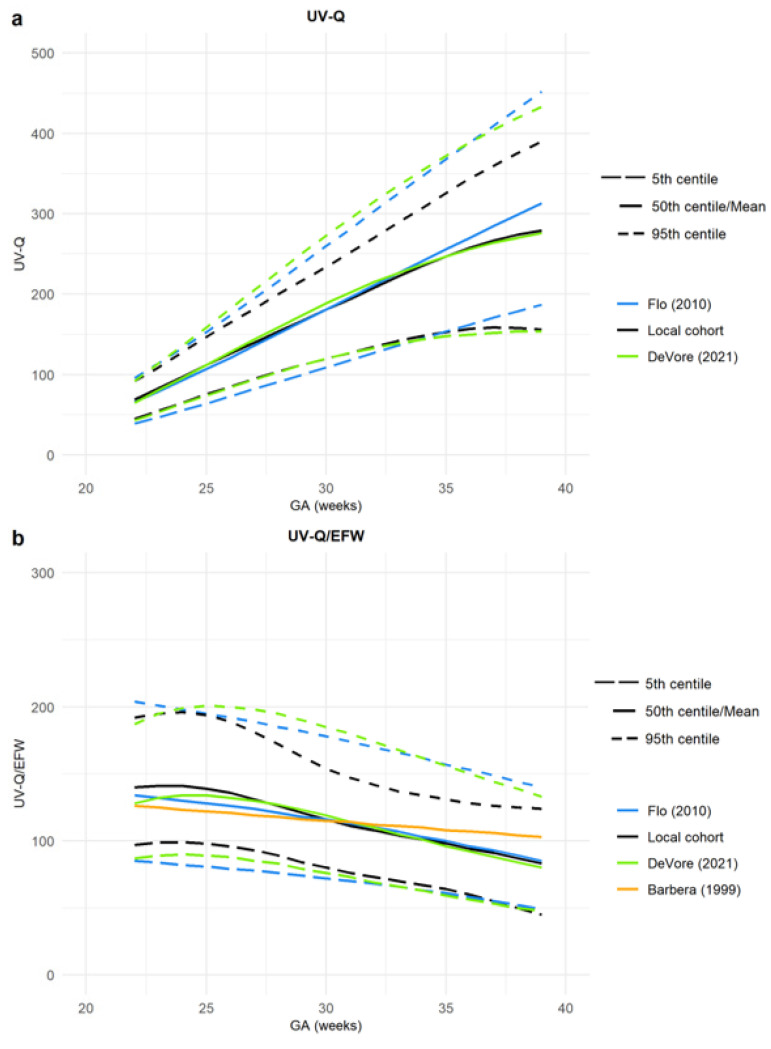
(**a**,**b**) Umbilical vein blood flow (UV-Q) 5th, 50th, and 95th percentiles, both (**a**) absolute and (**b**) normalized for estimated fetal weight (UV-Q/EFW) computed with the same methodology (i.e., on a free-floating loop of umbilical vein, in low-risk population, and with the same formula). Different colors represent the first author’s name and the year of publication [3,28,33].

In order to detect the deviations among available curves, we used our cohort as the reference, and we computed z-scores for all UV-Q and UV-Q/EFW computed with the same methodology (i.e., sampled at an FF portion of the UV and computed with the same formulae) (Table 4). Figure 6 represents UV-Q and UV-Q/EFW central values in low-risk populations sampled on an FF umbilical vein and computed with our same formula for UV-Q [3,28,33].

## 4. Discussion

### 4.1. The Main Findings of the Study

We found a substantial heterogeneity among UV-Q and UV-Q/EFW central values reported from 1990 to the present, selected by the robust standard procedures required for systematic reviews. We report our reference ranges for UV-Q, UV-Q/EFW, and UV-Q/AC, combined and sex-specific sampled at an FF UV portion. Part of the heterogeneity seems to be attributable to the sampling site used to compute UV-Q. In fact, the reported central values for the UV-Q sampled at FF UV portions were overall lower than those sampled on IA portions. When UV-Q central values computed with the same methodology as ours [28,33] were compared, there was an impressive overlap. Particularly, when comparing exclusively studies in which UV-Q calculations were performed using the same methodology [3,28,33] both in terms of population, sampling site, and formulae, we found an impressive overlap in central values. At 22 and 39 weeks, UV-Q corresponded to 66.6 ± 2.1 and 289.3 ± 20.5 mL/min, while UV-Q/EFW corresponded to 132.0 ± 6.2 to 87.7 ± 10.3 mL/min/kg, with an overall %err of 7%. These findings suggest that the assessment of UV-Q, assessed with the same methodology, may be reproducible and accurate, which opens a window of possible clinical applications.

### 4.2. Comment

Heterogeneity among reference ranges used in perinatology is not a novelty [44], and it has been reported for fetal biometry, Doppler velocimetry, and birthweight [44,45,46]. Reasons behind this heterogeneity are numerous and are beyond the scope of this manuscript. Despite the reported heterogeneity, most of these are used for clinical management in everyday practice.

Our systematic review highlights substantial differences among available reference charts for UV-Q and UV-Q/EFW that might be referred to various technological and methodological discrepancies. Some of the heterogeneity may be also related to the study design; most studies are cross-sectional [3,18,28,34,36,37,38,39,40,41,42,43], while relatively fewer are longitudinal studies [33,35]. Moreover, part of the heterogeneity seems to be related to the sampling site. UV-Q sampled at the IA portion of the UV showed greater values than those sampled at the FF portion and a greater dispersion among the reported values. The values reported in the low-risk population for UV-Q and UV-Q/EFW sampled at the IA portion are 80 ± 31 mL/min and 330 ± 112 mL/min at 22 weeks of gestation, and 125 ± 55 mL/min/kg and 73 ± 22 mL/min/kg at 39 weeks of gestation (%err of 36%), respectively [34,35,39,40,41,42,43]. These values sampled at the FF portion, at 22 and 39 weeks of gestation, are 62 ± 8 mL/min and 304 ± 63 mL/min for UV-Q and 130 ± 10 mL/min/kg and 93 ± 18 mL/min/kg for UV-Q/EFW (%err of 15%), respectively [18,28,33,36,37,39,42]. Part of the heterogeneity might also be attributed to the different sample sizes of the studies and different statistical procedures used to assess the central values and their percentiles.

#### 4.2.1. Methodology

Theoretically, the blood flow measured through the UV should be parabolic, regardless of the measurement site. However, in practice, this concept does not apply, while it has been shown that the velocity distribution coefficient changes according to the sampling site [47], affecting the evaluation of UV-Q. Thus, the choice of the sampling site is important and should be made a priori in order to make the results comparable. The preference of IA over the FF sampling site, and vice versa, has some argumentations. The main advantage of using the IA portion is its fixed location, thus making the reproducibility of the sampling site apparently easier. The angle of insonation is of crucial importance for the accurate measurement of UV-Q, and it should be maintained close to 0° for precise flow velocity evaluation. Fixed fetal position, bone shadowing, and other technical aspects, especially toward term, might represent an important obstacle of IA evaluation. In addition, flow velocity at the umbilical inlet is affected by possible turbulences caused by the umbilical ring and the change of diameter along the amniotic portion to the intra-hepatic portion of the vein. These are easily overcome by UV-Q assessment at the FF portion. On the other side, due to its length and mobility, doubts have been raised regarding the reliability of repeated measurements from the FF portion [48].

Despite the difficulties in standardizing the sampling site, measurements obtained at the FF portion proved to be reproducible, especially when performed far from the placental insertion [2,3]. The use of the FF portion of the umbilical cord for UV-Q measurement has been validated by Galan et al. [49] in an animal model and more recently by Figueras et al. [19], thus being a candidate for methodological recommendation. Sampling at the UV FF portion has another advantage. UV-Q is a measure based on the formula: *UV-Q* = *CSA* × mean velocity × 60, where flow is expressed in mL/min, the CSA is the cross-sectional area expressed in mm^2^, and the mean velocity is computed as mm/s. When UV-Q is evaluated at the FF portion, it is also possible to evaluate the CSA directly [32,37] or indirectly from the measurement of the diameters [3,6] through the formula: CSA = π × (diameter/2)^2^. The radius-squared itself represents a source of inaccuracy, as any error in its measurement is squared and amplified. To minimize this error, it is recommended to use an average of 3 to 5 successive measurements on a straight segment of the UV [50].

Moreover, ultrasound software extracts the mean velocity from the instantaneous Doppler shift analysis as an intensity-weighted mean velocity (IVmean). This creates a second source of inaccuracy, especially when peak velocities are relatively slow (14–18 cm/s from 20 to 38 weeks of gestation). The ideal flow model is a perfect parabolic flow uniformly distributed in the lumen of vessel. The mean velocity can be calculated by adding a correction coefficient which considers the ideal flow shape in the CSA, thus using the following formula:

mean velocity=TAMXV×0.5

Consequently, it is not only the sampling site that is relevant but the whole methodology that allows the calculation of the blood flow volume, which also depends on the ultrasound equipment.

#### 4.2.2. Experimental Research

In vivo models have been used for assessing the accuracy of umbilical vein blood flow. Doppler measurements of umbilical venous blood flow have been found to be accurate when compared with several gold standards for in vivo flow calculation. The simple “diameter-peak velocity × 0.5” methodology was applied to two veins of fetal lambs versus historical measurements of flow obtained with invasive techniques [3]. Gestational ages and fetal weights were not different between the animals studied (129.6 ± 2.8 days, 2.75 ± 0.26 kg, respectively) and the steady-state data (131.6 ± 4.1 days, 2.94 ± 0.68, respectively). A study by Galan et al. [48] was then performed in an ovine model [48]. Their results showed that umbilical venous blood flow volume determined by triplex-mode ultrasonography differed by less than 1% from the true flow measurement obtained by the steady-state diffusion technique (207 ± 9 vs. 208 ± 7 mL/min/kg).

### 4.3. Strengths and Limitations of the Study

We used the best available methodology and predefined protocol to perform this systematic review, allowing for an objective quality evaluation of the studies. We aimed to perform an objective comparison and to quantify the differences among the reported reference ranges, but the lack of some crucial statistical data made this evaluation not feasible. This is the rationale behind the decision to use our data as a reference point and to express the distance between the curves in terms of z-scores instead of performing only a visive, subjective evaluation. It has to be acknowledged that the calculation involved values derived from different estimation methods for the construction of the curves (i.e., best fits, median, or mean). For these reasons, these z-scores should be carefully evaluated. 

The fact that we used our cohort as the reference point made the comparison with UV reference ranges obtained on the IA portion impossible. Thus, our conclusions are mainly related to the UV-Q assessment on the UV FF portion.

Similarly to other authors [3,28,33], we provided an automatic UV-Q and UV-Q/EFW calculator as well as respective z-scores and percentiles for specific gestational weeks.

## 5. Conclusions

The measurement of UV-Q defines the blood flow volume delivered from the placenta to the fetus. The clinical value of UV-Q in understanding fetal adaptation to poor oxygenation and hypo-nutrition seems intuitive. However, its application, both in research and clinical settings, is still underestimated, mainly due to the concerns and criticisms regarding the measurement accuracy and reproducibility [16]. Our data suggest that when the same methodology is used for the UV-Q calculation, reproducible values may be obtained in line with other studies [19,32,37]. These findings should encourage further investigation on the potential role of UV-Q.

## Figures and Tables

**Table 1:** Main characteristics and results of the included studies, divided according to the sampling site (i.e., intra-abdominal [IA], free floating [FF]) and the formula used to compute umbilical vein blood flow volume (UV-Q).

	First Author, Year of Publication	Study Type	Population (N)	Gestational Age	Description of Population	Available Percentiles	Formula for Q-UV Measurement	Mean Values of Q UV (mL/min)	Mean Values of Q-UV Normalized to EFW (mL/min/kg)
**Free-floating portion**									
**1**	DeVore et al., 2021 [28]	Prospective cross-sectional study	240	20 to 40 weeks	Singleton low-risk pregnancies	Yes	0.5 × TaMXV × π(D/2)^2^ × 60		
**2**	Flo et al., 2010 [33]	Prospective longitudinal study	53	22 to 39^+6^ weeks	Singleton low-risk pregnancies	Yes	Two formulae used separately: (1) 0.5 × TaMXV × π(D/2)^2^ × 60 (2) Vwmean × π(D/2)^2^ × 60	1st formula: from 53 to 250 (22–39^+6^ weeks) 2nd formula: from 66 to 313 (22–39^+6^ weeks)	1st formula: from 110 to 68 (22–39^+6^ weeks)
**3**	Boito et al., 2003 [36]	Cross-sectional matched control study	64	18 to 36 weeks	32 low-risk ^£^ and 32 diabetic women	No	0.06 × TaMXV × π × (D/2)^2^	100.5 (in diabetic women) versus 106.2 (in controls)	94.2 (in diabetic women) versus 109.4 (in controls)
**4**	Boito et al., 2002 [37]	Prospective cross-sectional study	133	20 to 36 weeks	100 low-risk ^£^ and 33 SGA	No	TaMXV × π × (D/2)^2^	33.2 (at 20 weeks) 221.0 (at 36 weeks)	117.5 (at 20 weeks) 78.3 (at 36 weeks)
**5**	Barbera et al., 1999 [3]	Prospective cross-sectional study	70	20 to 38 weeks	Singleton low-risk pregnancies	No	0.5 × TaMXV × π × (D/2)^2^ × 60	54 (at 23 weeks) 320 (at 38 weeks)	125 (at 23 weeks) 104 (at 38 weeks)
**6**	Sutton et al., 1990 [18]	Prospective cross-sectional study	74	19 to 42 weeks	Singleton low-risk pregnancies	No	FVI_UV_/s × π × (D/2)^2^ × 60		105–130
**Intra-abdominal portion**									
**7**	Rizzo et al., 2016 [40]	Prospective cross-sectional study	852	14 to 40 weeks	Singleton low-risk pregnancies	No	0.5 × TaMXV × π(D/2)^2^ × 60		
**8**	Tchirikov et al., 2009 [34]	Prospective cross-sectional study	181	17 to 41 weeks	148 low-risk and 33 with poor fetal outcome	No	TaMXV × π × (D/2)^2^	160.2 (in compromised fetuses) versus 253.3 (in controls)	115.1 (in compromised fetuses) versus 200.3 (in controls)
**9**	Acharya et al., 2005 [35]	Prospective longitudinal study	130	19 to 42 weeks	Singleton low-risk pregnancies	Yes	Two formulae used separately: (1) 0.5 × Vmax × π(D/2)^2^ × 60 (2) Vwmean × π(D/2)^2^ × 60	1st formula: from 27.6 to 271.1 (19–41 weeks) 2nd formula: from 27.13 to 273.4 (19–41 weeks)	1st formula: from 74.7 to 63.2 (19–41 weeks) 2nd formula: from 73.5 to 63.3 (19–41 weeks)
**10**	Tchirikov et al., 2002 [38]	Retrospective, cross- sectional clinical study	85	17 to 41 weeks	of whom 15 had poor fetal outcomes	No	iVmean × π(D/2)^2^	17 *	−2.2 **
**11**	Kiserud et al., 2000 [41]	Prospective cross-sectional study	197	18 to 41 weeks	Singleton low-risk pregnancies	No	Vwmean × π × (D/2)^2^		
**12**	Tchirikov et al., 1998 [43]	Prospective cross-sectional study	−75 (singleton) −10 (twin pregnancies)	100 to 300 days	Singleton: −55 low-risk ^£^ −20 FGR	No			
**Both at free-floating and intra-abdominal portions**									
**13**	Wang et al., 2021 [39]	Prospective cross-sectional study	907	20 to 39 weeks	Singleton low-risk pregnancies	No	iVmean × 60 × π(D/2)^2^	FL: from 32.6 to 381.9 (20–39 weeks) IA: from 31.5 to 360.1 (20–39 weeks)	
**14**	Bellotti et al., 2000 [42]	Prospective cross-sectional study	137	20 to 38 weeks	Singleton low-risk pregnancies	No	0.5 × TaMXV × π × (D/2)^2^		

Blank cells correspond to data not provided by the authors. Green lines correspond to authors using our same methodology in the computing of UV-Q, both in terms of sampling site and formula used. D, diameter; FF, free floating; FVI, flow velocity integral; IA, intra-abdominal; iVmean, intensity-weighted mean velocity; Q-UV, umbilical vein blood flow volume; SD, standard deviation; SGA, small for gestational age; TaMXV, time averaged mean velocity; Vmax, maximum velocity; Vmean, mean velocity; Vwmean, weighted mean velocity. * mL/min/week. ** mL/min/kg/weeksexpressed as the mean of three successive measurements of the inner diameter of the vessel, ^£^ UV-Q curve returned by the authors is based only on low-risk pregnant women.

**Table 2:** Demographic, obstetric, and neonatal characteristics of the cohort. Data are represented as number and percentage, mean ± standard deviation, or median with interquartile range, as appropriate.

	Population (*n* = 255)
**Maternal age (years)**	33 (29–36)
**Non-Caucasian ethnicity**	3 (1.2%)
**Maternal pre-pregnancy BMI (kg/m^2^)**	22 (20–24)
**Nulliparous**	122 (47.7%)
**EFW percentile**	50 (36–63)
**GA at delivery**	40^+3^ (39^+1^–40^+5^)
**Birthweight (g)**	3420 (3175–3645)
**Male fetuses**	133 (52.2%)

BMI, body mass index; EFW, estimated fetal weight; GA, gestational age.

**Table 3:** The 5th, 10th, 50th, 90th, and 95th percentiles for UV-Q, UV-Q/EFW, and UV-Q/CA at each gestational week are represented. Sex-combined percentiles are represented.

	UV-Q					UV-Q/EFW					UV-Q/AC				
GA (Weeks)	5th	10th	50th	90th	95th	5th	10th	50th	90th	95th	5th	10th	50th	90th	95th
20	28	31	45	60	64	92	101	137	174	51	0.19	0.21	0.30	0.41	0.44
21	36	40	57	75	80	95	103	139	178	66	0.21	0.24	0.35	0.47	0.51
22	45	50	69	92	98	97	105	140	181	95	0.24	0.27	0.39	0.53	0.57
23	55	60	83	109	117	99	106	141	183	112	0.27	0.30	0.43	0.59	0.63
24	65	71	97	128	136	99	106	141	184	132	0.29	0.33	0.48	0.64	0.70
25	76	82	112	147	156	98	105	139	182	154	0.32	0.36	0.52	0.70	0.75
26	85	93	129	165	175	96	103	136	177	176	0.34	0.38	0.55	0.75	0.81
27	95	103	140	182	194	93	99	131	171	198	0.36	0.41	0.59	0.80	0.86
28	104	113	154	200	212	89	95	126	163	220	0.38	0.43	0.62	0.84	0.91
29	112	122	167	217	230	84	91	121	154	241	0.40	0.45	0.65	0.88	0.96
30	120	131	181	234	248	80	87	116	147	261	0.42	0.47	0.68	0.92	1.00
31	127	140	194	252	267	76	83	111	140	281	0.44	0.49	0.71	0.96	1.04
32	135	149	208	270	286	73	80	108	135	300	0.45	0.51	0.73	0.99	1.07
33	142	158	222	289	307	70	77	104	131	319	0.47	0.53	0.76	1.03	1.11
34	148	166	235	307	328	67	74	101	127	337	0.48	0.54	0.78	1.05	1.14
35	153	173	247	326	349	64	71	98	124	355	0.49	0.55	0.80	1.08	1.17
36	157	178	258	344	369	60	67	94	121	371	0.50	0.57	0.81	1.1	1.19
37	159	182	267	360	389	55	62	91	119	385	0.51	0.58	0.83	1.12	1.21
38	158	184	274	376	409	50	57	87	117	397	0.52	0.59	0.84	1.14	1.23
39	156	183	279	389	428	45	53	83	115	406	0.53	0.60	0.86	1.16	1.25
40	150	181	282	402	446	39	48	80	113	415	0.54	0.60	0.87	1.17	1.27
41	143	176	283	412	464	34	43	76	111	422	0.54	0.61	0.88	1.19	1.29

**Table 4:** Standardized scores for umbilical vein blood flow volume (UV-Q) and UV-Q normalized for estimated fetal weight (UV-Q/EFW) on free-floating umbilical vein calculated using our cohort as the reference. Values in bold italics represent z-scores in the interval [−1, 1].

	UV-Q							UV-Q/EFW				
GA (Weeks)	Sutton 1990	Bellotti 2000	Boito 2003	Flo 2010	DeVore 2021	Wang 2021	GA (Weeks)	Sutton 1990	Barbera 1999	Bellotti 2000	Flo 2010	DeVore 2021
22	***−0.56***	−1.92	***−0.67***	***−0.25***	***−0.33***	***−0.34***	22	***0.32***	***−0.65***	***−0.79***	***−0.28***	***−0.56***
23	***−0.60***	−1.36	***−0.50***	***−0.18***	***−0.09***	***0.04***	23	***0.13***	***−0.45***	***−0.56***	***−0.25***	***−0.25***
24	***−0.68***	−1.32	***−0.50***	***−0.14***	***−0.04***	***0.21***	24	***0.07***	***−0.45***	***−0.52***	***−0.27***	***−0.17***
25	*−1.18*	−2.10	***−0.83***	***−0.24***	***0.00***	***0.48***	25	***0.13***	***−0.63***	***−0.74***	***−0.41***	***−0.18***
26	*−1.05*	−1.85	***−0.75***	***−0.19***	***0.07***	***0.56***	26	***0.21***	***−0.63***	***−0.79***	***−0.42***	***−0.17***
27	*−1.02*	−1.80	***−0.76***	***−0.13***	***0.13***	***0.67***	27	***0.39***	***−0.55***	***−0.69***	***−0.32***	***−0.05***
28	***−0.86***	−1.57	***−0.70***	***−0.08***	***0.14***	***0.68***	28	***0.40***	***−0.27***	***−0.37***	***−0.17***	***0.03***
29	***−0.69***	−1.39	***−0.63***	***−0.02***	***0.16***	***0.70***	29	***0.64***	***−0.21***	***−0.29***	***−0.12***	***0.08***
30	***−0.61***	−1.39	***−0.66***	***0.00***	***0.17***	***0.78***	30	***0.76***	***−0.04***	***−0.12***	***0.00***	***0.12***
31	***−0.53***	−1.49	***−0.75***	***0.05***	***0.19***	***0.98***	31	1.07	***0.14***	***0.05***	***0.10***	***0.14***
32	***−0.39***	−1.55	***−0.83***	***0.07***	***0.17***	1.12	32	1.67	***0.28***	***0.21***	***0.14***	***0.14***
33	***−0.14***	−1.18	***−0.68***	***0.07***	***0.07***	***0.95***	33	1.09	***0.29***	***0.25***	***0.12***	***0.04***
34	***0.10***	−1.36	***−0.84***	***0.13***	***0.04***	1.23	34	1.55	***0.50***	***0.44***	***0.11***	***0.00***
35	***0.43***	−1.20	***−0.80***	***0.18***	***0.00***	1.30	35	1.72	***0.58***	***0.64***	***0.12***	***−0.12***
36	***0.53***	***−0.69***	***−0.50***	***0.15***	***−0.03***	***0.91***	36	1.33	***0.54***	***0.58***	***0.08***	***−0.08***
37	***1.00***	***−0.68***	***−0.55***	***0.26***	***−0.04***	1.20	37	1.42	***0.64***	***0.68***	***0.08***	***−0.13***
38	1.79	***−0.63***	***−0.59***	***0.44***	***−0.07***	1.67	38	1.89	***0.89***	***1.00***	***0.11***	***−0.16***
39	1.75	***−0.27***	***−0.34***	***0.42***	***−0.04***	1.35	39	1.67	***0.87***	***1.00***	***0.09***	***−0.13***

## Data Availability

The data presented in this study are available on request from the corresponding author.

## Supplementary Materials

The following supporting information can be downloaded at: https://www.mdpi.com/article/10.3390/jcm12093132/s1. Figure S1. Risk of bias author’s judgement review based on Quality Assessment of Diagnostic Accuracy Studies Tool (QUADAS). (a) Risk of bias in each domain are presented as percentages across included studies. Green, low risk of bias; red, high risk of bias; yellow, some concerns of bias; blue, unclear risk of bias. (b) Concerns regarding applicability. Figure S2: The Figure represents umbilical vein blood flow volume (UV-Q) central values in low-risk and general population in studies that investigated UV-Q in an intra-abdominal (IA) portion of the umbilical vein: (a) UV-Q absolute value; and (b) normalized for estimated fetal weight (UV-Q/EFW). Different colors represent the first author’s name and the year of publication [34,35,38,39,40,41,42,43]. Figure S3: The Figure represents umbilical vein blood flow volume (UV-Q) central values in low-risk and general population in studies that investigated UV-Q on a free-floating (FF) portion of the umbilical cord: (a) UV-Q absolute value; and (b) normalized for estimated fetal weight (UV-Q/EFW). Different colors represent the first author’s name and the year of publication [3,18,28,33,36,37,39,42]. Table S1. Literature search strategy in Pubmed and Scopus. Table S2. The Table represents the 5th, 10th, 50th, 90th and 95th sex-specific percentiles for umbilical vein blood flow volume (UV-Q), UV-Q normalized for estimated fetal weight (UV-Q/EFW) and UV-Q normalized for the abdominal circumference (UV-Q/AC) from our local cohort. Table S3. The Table represents the central values for umbilical vein blood flow volume (UV-Q) of each manuscript included in this systematic review. Table S4. The Table represents the central values for umbilical vein blood flow volume normalized for estimated fetal weight (UV-Q/EFW) of each manuscript included in this systematic review.
