# Supplementary material for: Umbilical Vein Blood Flow in Uncomplicated Pregnancies: Systematic Review of Available Reference Charts and Comparison with a New Cohort
Source: J Clin Med. 2023 Apr 26;12(9):3132. doi: 10.3390/jcm12093132 (PMC10179232; doi:10.3390/jcm12093132)
Supplement: Supplementary file 1 [file jcm-12-03132-s001.zip › jcm-2330131-supplementary.pdf]

Supplementary material for the manuscript

"Umbilical vein blood flow in uncomplicated pregnancies: systematic review of available reference charts and comparison with a new cohort"

Moira Barbieri[1], Giulia Zamagni[2], Ilaria Fantasia[1], Lorenzo Monasta[2], Leila Lo Bello[1], Mariachiara Quadrifoglio[1], Giuseppe Ricci[1,3], Gianpaolo Maso[1], Monica Piccoli[1], Daniela D. Di Martino[4], Enrico M. Ferrazzi[4,5], Tamara Stampalija[1,3,*]

Figure S1. Risk of bias author's judgement review based on Quality Assessment of Diagnostic Accuracy Studies Tool (QUADAS). (a) Risk of bias in each domain are presented as percentages across included studies. *Green, low risk of bias; red, high risk of bias; yellow, some concerns of bias; blue, unclear risk of bias.* (b) Concerns regarding applicability.

a)

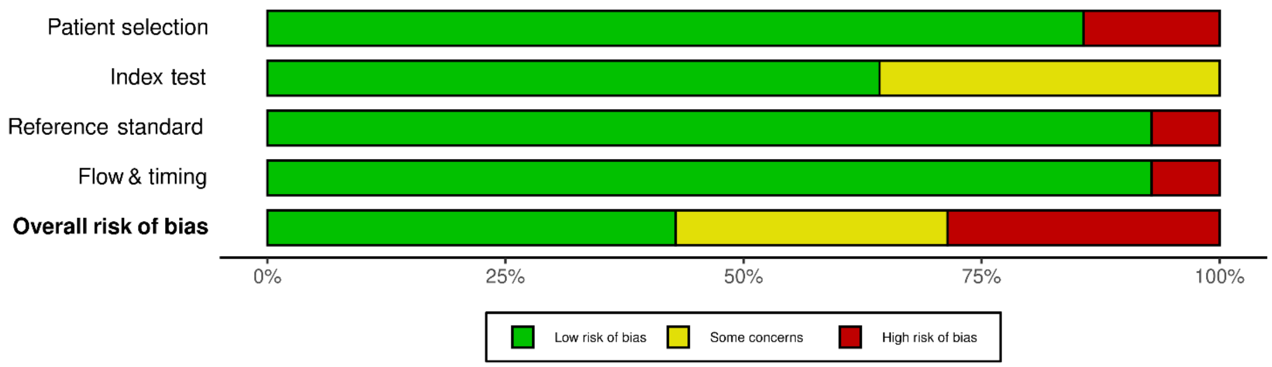

b)

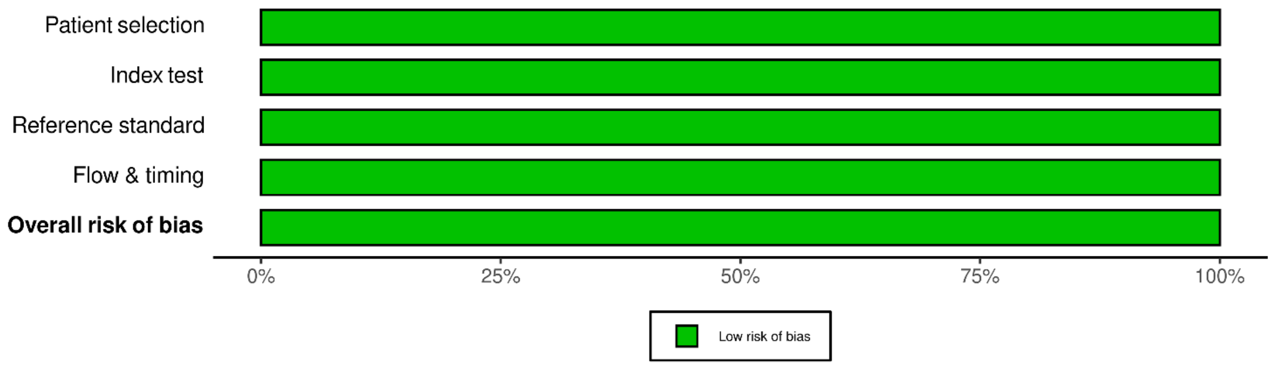

Figure S2: The Figure represents umbilical vein blood flow volume (UV-Q) central values in low-risk and general population in studies that investigated UV-Q in an intra-abdominal (IA) portion of the umbilical vein: a) UV-Q absolute value; and b) normalized for estimated fetal weight (UV-Q/EFW). Different colors represent the first author's name and the year of publication.

a)

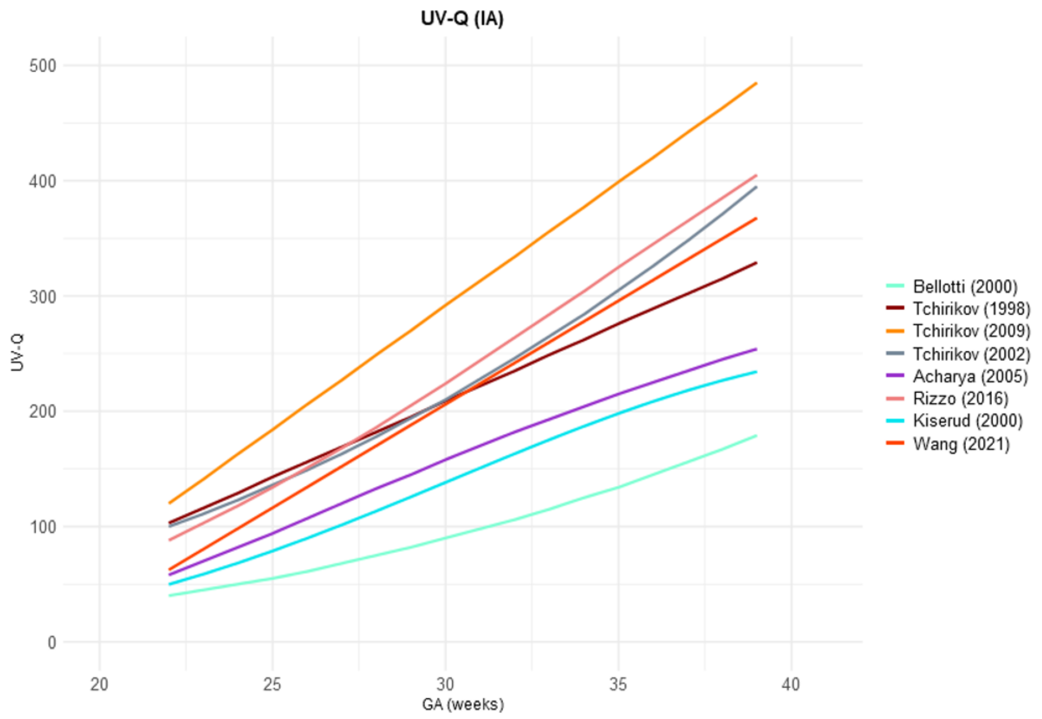

b)

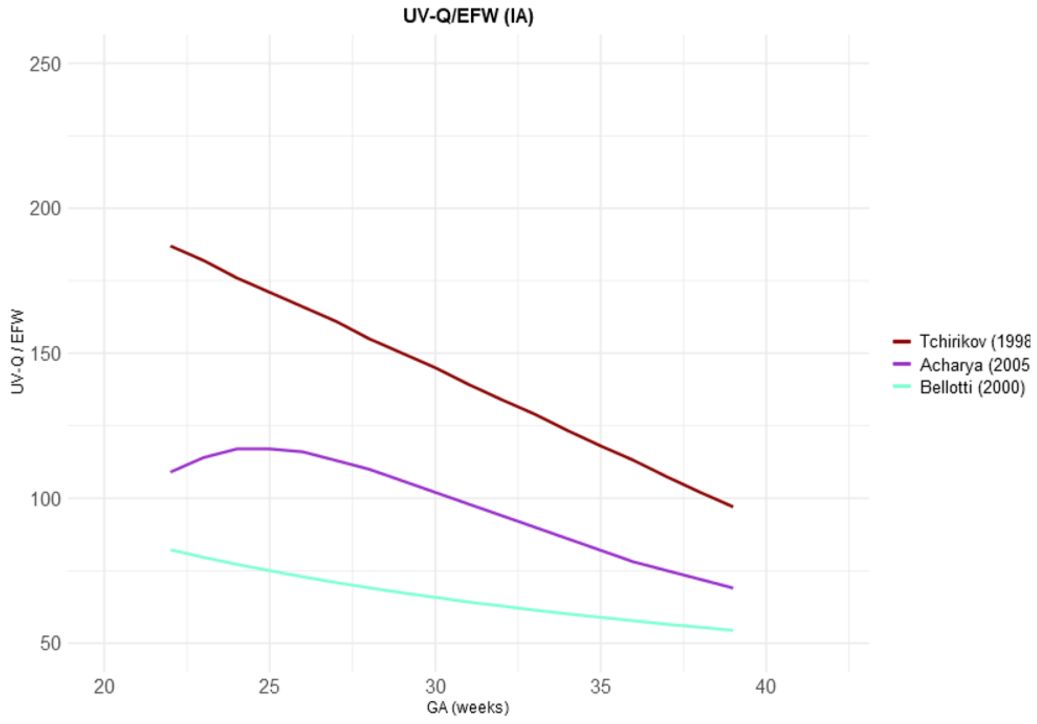

Figure S3: The Figure represents umbilical vein blood flow volume (UV-Q) central values in low-risk and general population in studies that investigated UV-Q on a free-floating (FF) portion of the umbilical cord: a) UV-Q absolute value; and b) normalized for estimated fetal weight (UV-Q/EFW). Different colors represent the first author's name and the year of publication.

a)

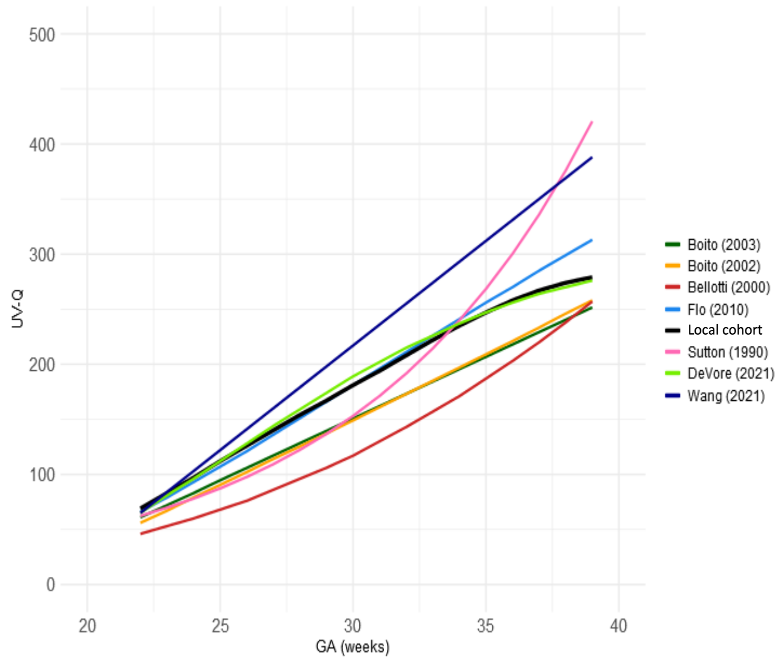

b)

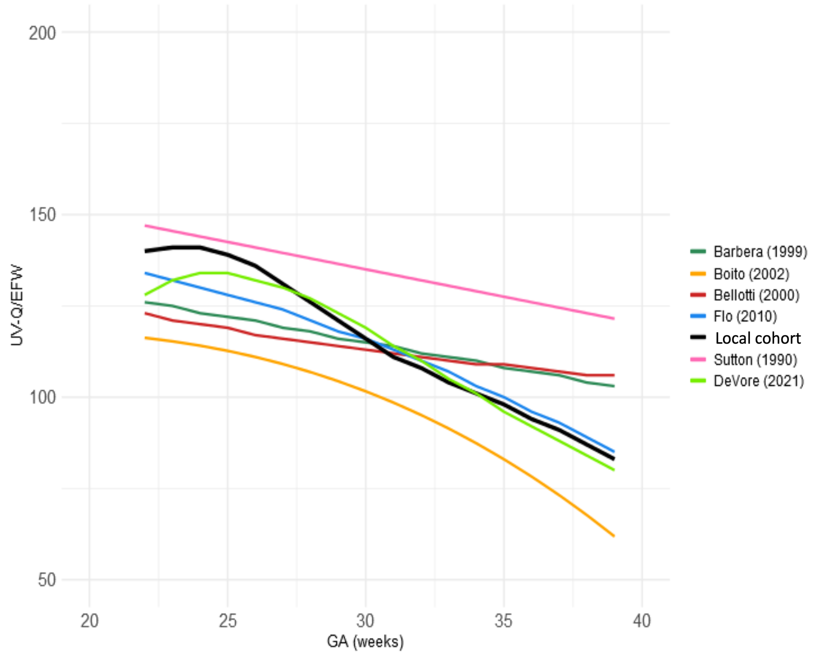

Table S1. Literature search strategy in Pubmed and Scopus.

We ran the following search strategy in **PubMed** and **Scopus** until December 2021:

Library	Search terms	Results
PubMed	(umbilical vein blood flow) OR (umbilical venous blood flow) AND (volume)	283
Scopus	(TITLE-ABS-KEY(umbilical vein blood flow) OR TITLE-ABS-KEY(umbilical venous blood flow) AND TITLE-ABS-KEY(volume)) AND (LIMIT-TO (SUBJAREA,"MEDI"))	304

Table S2. The Table represents the 5th, 10th, 50th, 90th and 95th sex-specific percentiles for umbilical vein blood flow volume (UV-Q), UV-Q normalized for estimated fetal weight (UV-Q/EFW) and UV-Q normalized for the abdominal circumference (UV-Q/AC) from our local cohort.

	UV-Q										UV-Q/EFW										UV-Q/AC									
GA (week)	Female fetuses					Male fetuses					Female fetuses					Male fetuses					Female fetuses					Male fetuses				
	5th	10th	50th	90th	95th	5th	10th	50th	90th	95th	5th	10th	50th	90th	95th	5th	10th	50th	90th	95th	5th	10th	50th	90th	95th	5th	10th	50th	90th	95th
20	17	29	41	52	37	17	29	41	52	37	101	109	142	183	197	91	100	134	173	185	0.19	0.22	0.29	0.39	0.43	0.18	0.21	0.31	0.41	0.44
21	25	38	55	70	55	25	38	55	70	55	101	109	142	184	197	93	101	135	174	186	0.23	0.25	0.34	0.46	0.51	0.21	0.24	0.35	0.46	0.50
22	34	49	69	89	74	34	49	69	89	74	101	109	142	184	198	94	102	136	175	187	0.26	0.29	0.39	0.52	0.58	0.23	0.27	0.39	0.52	0.56
23	43	61	85	110	94	43	61	85	110	94	101	109	142	184	197	94	103	136	176	188	0.30	0.33	0.44	0.59	0.66	0.26	0.30	0.43	0.57	0.62
24	54	72	101	130	117	54	72	101	130	117	100	108	141	182	195	94	102	136	177	189	0.33	0.36	0.49	0.66	0.73	0.28	0.33	0.47	0.62	0.68
25	65	83	114	148	138	65	83	114	148	138	98	106	138	178	191	92	101	135	176	189	0.36	0.39	0.53	0.71	0.79	0.31	0.35	0.51	0.68	0.73
26	74	92	126	165	158	74	92	126	165	158	95	102	133	172	184	90	98	133	174	186	0.38	0.42	0.57	0.76	0.84	0.33	0.38	0.55	0.73	0.79
27	83	100	137	180	176	83	100	137	180	176	91	98	127	164	176	86	95	130	170	182	0.40	0.45	0.60	0.81	0.89	0.35	0.40	0.58	0.77	0.84
28	91	109	145	194	193	91	109	145	194	193	86	93	121	156	167	83	91	126	165	177	0.42	0.47	0.63	0.85	0.94	0.37	0.43	0.61	0.82	0.88
29	99	117	159	210	209	99	117	159	210	209	82	89	115	148	159	78	87	121	159	170	0.44	0.49	0.66	0.89	0.98	0.39	0.45	0.64	0.86	0.93
30	107	128	172	229	227	107	128	172	229	227	79	85	111	142	153	74	83	117	153	163	0.47	0.51	0.69	0.93	1.03	0.41	0.47	0.67	0.90	0.97
31	117	140	188	251	248	117	140	188	251	248	78	84	108	140	150	70	79	112	147	157	0.49	0.54	0.73	0.98	1.08	0.42	0.49	0.70	0.93	1.01
32	129	154	205	275	272	129	154	205	275	272	77	83	108	139	149	67	76	108	141	150	0.51	0.56	0.76	1.02	1.13	0.44	0.50	0.72	0.96	1.04
33	142	168	223	300	299	142	168	223	300	299	76	82	107	138	148	64	73	104	135	144	0.53	0.59	0.79	1.07	1.18	0.45	0.52	0.75	0.99	1.07
34	155	180	240	323	327	155	180	240	323	327	74	80	105	136	146	61	70	99	129	138	0.55	0.61	0.82	1.10	1.22	0.46	0.53	0.77	1.02	1.11
35	166	189	255	343	351	166	189	255	343	351	70	77	102	133	143	58	66	95	124	132	0.56	0.62	0.83	1.12	1.24	0.48	0.55	0.79	1.05	1.13
36	174	197	269	363	373	174	197	269	363	373	66	73	99	130	139	55	63	90	118	126	0.56	0.62	0.84	1.13	1.25	0.49	0.56	0.80	1.07	1.16
37	180	201	281	381	394	180	201	281	381	394	62	68	95	127	136	52	59	85	112	119	0.56	0.62	0.83	1.12	1.24	0.50	0.57	0.82	1.10	1.19
38	182	204	294	401	414	182	204	294	401	414	56	64	92	124	134	49	56	80	105	112	0.55	0.61	0.82	1.10	1.22	0.51	0.58	0.84	1.12	1.21
39	181	203	306	421	434	181	203	306	421	434	51	59	90	123	132	47	53	76	99	106	0.54	0.59	0.80	1.08	1.19	0.52	0.60	0.86	1.14	1.24
40	177	200	318	441	455	177	200	318	441	455	44	54	88	121	130	44	50	71	93	99	0.52	0.57	0.77	1.04	1.15	0.53	0.61	0.88	1.17	1.26
41	167	193	331	461	476	167	193	331	461	476	38	49	86	119	128	42	47	66	86	92	0.50	0.55	0.75	1.00	1.11	0.54	0.62	0.89	1.19	1.29

Table S3. The Table represents the central values for umbilical vein blood flow volume (UV-Q) of each manuscript included in this systematic review.

	UV-Q															
	Free-floating portion								Intra-abdominal portion							
GA (weeks)	LOCAL COHORT	Wang 2021 (FL)	DeVore 2021	Flo 2010	Boito 2003	Boito 2002	Bellotti 2000 (FL)	Sutton 1990	Wang 2021 (IA)	Rizzo 2016	Tchirikov 2009	Acharya 2005	Tchirikov 2002	Bellotti 2000 (IA)	Kiserud 2000	Tchirikov 1998
**22**	69	65	65	66	61	56	46	62	62	88	120	58	100	40	50	103
**23**	83	84	81	79	72	67	53	70	80	103	141	70	111	45	59	116
**24**	97	103	96	93	83	79	60	78	98	118	163	82	123	50	68	129
**25**	112	122	112	107	95	90	68	87	116	134	184	94	136	55	79	143
**26**	126	141	128	121	106	102	76	98	134	151	206	107	149	61	90	156
**27**	140	160	144	136	117	114	86	109	152	168	227	120	163	68	101	169
**28**	154	179	159	151	128	125	96	122	170	186	249	133	178	75	113	182
**29**	167	198	174	166	139	137	106	137	188	205	270	145	194	82	126	195
**30**	181	217	189	181	151	149	117	153	206	224	292	158	210	90	138	209
**31**	194	236	202	196	162	161	130	171	224	244	313	170	228	98	151	222
**32**	208	255	215	211	173	173	143	192	242	264	334	182	246	106	163	235
**33**	222	274	226	226	184	185	157	214	260	284	356	193	265	115	175	249
**34**	235	293	237	241	196	197	171	240	278	304	377	204	284	125	187	262
**35**	247	312	247	256	207	209	187	268	296	325	399	215	305	134	198	276
**36**	258	331	256	270	218	221	203	300	314	345	420	225	326	145	209	289
**37**	267	350	264	285	229	233	220	336	332	365	442	235	348	156	218	302
**38**	274	369	270	299	240	246	238	376	350	385	463	245	371	167	227	315
**39**	279	388	276	313	252	258	257	421	368	405	485	254	395	179	234	329

Table S4. The Table represents the central values for umbilical vein blood flow volume normalized for estimated fetal weight (UV-Q/EFW) of each manuscript included in this systematic review.

	UV-Q/EFW									
	Free-floating portion							Intra-abdominal portion		
GA (weeks)	LOCAL COHORT	DeVore 2021	Flo 2010	Boito 2002	Bellotti 2000 (FL)	Barbera 2000	Sutton 1990	Acharya 2005	Tchirikov 1998	Bellotti 2000 (IA)
**22**	140	128	66	116	123	126	147	109	187	82
**23**	141	132	79	115	121	125	145.5	114	182	80
**24**	141	134	93	114	120	123	144	117	176	77
**25**	139	134	107	113	119	122	142.5	117	171	75
**26**	136	132	121	111	117	121	141	116	166	73
**27**	131	130	136	109	116	119	139.5	113	161	71
**28**	126	127	151	107	115	118	138	110	155	69
**29**	121	123	166	104	114	116	136.5	106	150	67
**30**	116	119	181	102	113	115	135	102	145	66
**31**	111	114	196	99	112	114	133.5	98	139	64
**32**	108	110	211	95	111	112	132	94	134	63
**33**	104	105	226	91	110	111	130.5	90	129	61
**34**	101	101	241	87	109	110	129	86	123	60
**35**	98	96	256	83	109	108	127.5	82	118	59
**36**	94	92	270	78	108	107	126	78	113	58
**37**	91	88	285	73	107	106	124.5	75	107	57
**38**	87	84	299	68	106	104	123	72	102	55
**39**	83	80	313	62	106	103	121.5	69	97	54
